# Matrine Targets Intestinal *Lactobacillus acidophilus* to Inhibit Porcine Circovirus Type 2 Infection in Mice

**DOI:** 10.3390/ijms241511878

**Published:** 2023-07-25

**Authors:** Zhigang Cao, Xiaoya Ling, Panpan Sun, Xiaozhong Zheng, Hua Zhang, Jia Zhong, Wei Yin, Kuohai Fan, Yaogui Sun, Hongquan Li, Na Sun

**Affiliations:** 1 Shanxi Key Laboratory for Modernization of TCVM, College of Veterinary Medicine, Shanxi Agricultural University, Taigu 030801, China; 15235415738@163.com (Z.C.);; 2 Centre for Inflammation Research, Queen’s Medical Research Institute, The University of Edinburgh, Edinburgh EH16 4TJ, UK; 3 Laboratory Animal Center, Shanxi Agricultural University, Taigu 030801, China

**Keywords:** matrine, PCV2, FMT, *Cap*, *L. acidophilus*, *IL-1β*

## Abstract

Porcine circovirus type 2 (PCV2) has caused huge economic losses to the pig industry across the world. Matrine is a natural compound that has been shown to regulate intestinal flora and has anti-PCV2 activity in mouse models. PCV2 infection can lead to changes in intestinal flora. The intestinal flora has proved to be one of the important pharmacological targets of the active components of Traditional Chinese Medicine. This study aimed to determine whether matrine exerts anti-PCV2 effects by regulating intestinal flora. In this study, fecal microbiota transplantation (FMT) was used to evaluate the effect of matrine on the intestinal flora of PCV2-infected Kunming (KM) mice. The expression of the *Cap* gene in the liver and the ileum, the relative expression of *IL-1β* mRNA, and the *Lactobacillus acidophilus* (*L. acidophilus*) gene in the ileum of mice were determined by real-time quantitative polymerase chain reaction (qPCR). ELISA was used to analyze the content of secretory immunoglobulin A (SIgA) in small intestinal fluid. *L. acidophilus* was isolated and identified from the feces of KM mice in order to study its anti-PCV2 effect in vivo. The expression of the *Cap* gene in the liver and the ileum and the relative expression of *L. acidophilus* and *IL-1β* mRNA in the ileum were determined by qPCR. The results showed that matrine could reduce the relative expression of *IL-1β* mRNA by regulating intestinal flora, and that its pharmacological anti-PCV2 and effect may be related to *L. acidophilus*. *L. acidophilus* was successfully isolated and identified from the feces of KM mice. The in vivo experiment revealed that administration of *L. acidophilus* also reduced the relative expression of *IL-1β* mRNA, and that it had anti-PCV2 effects in PCV2-infected mice. It was found that matrine could regulate the abundance of *L. acidophilus* in the gut of mice to exert an anti-PCV2 effect and inhibit PCV2-induced inflammatory response.

## 1. Introduction

PCV2 is the primary pathogen of porcine circovirus associated disease (PCVAD) [1]. In taxonomy, it belongs to the Circoviridae family, Circovirus genus, and is one of the smallest known animal viruses. The PCV2 genome is a single-stranded, negative-stranded, circular DNA bound by covalent bonds. The whole genome of PCV2 contains 11 open reading frame (ORFs), including ORF1, ORF2, ORF3, and ORF4 [2]. PCV2 capsid (cap) protein is a viral nucleocapsid protein encoded by open reading frame 2 (ORF2) and contains specific antigenic determinants [3]. *Cap* is an important immunogenic protein of PCV2, which is involved in the virus replication and viral infection and transmission [4,5]. Pigs have a strong susceptibility to PCV2, which can lead to symptoms such as dyspnea and abortion [6], pathological changes such as liver injury [7], interstitial pneumonia [8], and changes in intestinal flora [9]. The reason for changes in intestinal flora is that PCV2 infection can cause diarrhea in pigs. In addition, PCV2 can cause lung lesions, and based on the Traditional Chinese Medicine theory, “the lung and the large intestine are interior-exteriorly co-related”, and the gut-lung axis theory. It can also be speculated that dyspnea of mammals may be accompanied by changes in the structure of intestinal flora. Since the first discovery of PCV2 in 1998 [10], the virus has gradually spread worldwide. PCV2 was first reported in China in 2000, and it spread rapidly across the country, causing huge economic losses to the pig industry.

Matrine is an alkaloid extracted from *Sophora flavescens*. A large number of studies have shown that matrine has anti-tumor [11], anti-inflammatory [12], anti-allergy [13], and other pharmacological effects. Our previous studies have found that matrine significantly inhibited PCV2 replication in PRRSV/PCV2 co-infected primary porcine alveolar macrophages (PAM) and mouse models [14]. In addition, preliminary research in our group has found that matrine can significantly affect the structure of gut microbiota in KM mice. It is worth noting that matrine significantly increased the abundance of *L. acidophilus*, enhanced the colonization of *L. acidophilus* in each intestinal segment, and caused differences in metabolic pathways such as glycan biosynthesis and metabolism, transport, and catabolism.

Intestinal flora is a microbial community that exists symbiotically within the intestinal tract of the body. It has a wide variety, and it is the second gene pool of animals [15]. A large number of studies have shown that intestinal flora is highly correlated with a variety of diseases, including tumors [16], inflammation [17], neurological diseases [18], and viral infections [19]. Research on intestinal flora has changed people’s understanding of diseases, especially viral diseases. Previous studies have shown that PCV2 infection can lead to changes in intestinal flora. After PRRSV/PCV2 co-infection, the abundance of *L. acidophilus* decreased in pigs with severe clinical symptoms [9]. *L. acidophilus* is one of the most important probiotics in the body. It has pharmacological effects, including antiviral [20], anti-inflammatory [21], and anti-tumor properties [22], and also inhibits harmful bacteria [23]. Studies have shown that intestinal flora is an important target for Traditional Chinese Medicine to exert its pharmacological effects [24,25]. It is suggested that matrine may regulate intestinal flora, especially *L. acidophilus*, to exert an anti-PCV2 effect. Therefore, in this study, fecal bacteria solution isolated from matrine-treated mice was transplanted to test whether matrine plays an anti-PCV2 role by regulating the intestinal flora in mice. We also isolate and culture *L. acidophilus* from mouse feces in order to evaluate the anti-PCV2 effect of *L. acidophilus* in mice, which allows us to explore the mechanism of matrine against PCV2.

## 2. Results

### 2.1. Matrine-Treated Fecal Bacterial Solution Reduced the Relative Expression of IL-1β mRNA and Promoted the Colonization of L. acidophilus, Indicating that It Has Anti-PCV2 Effects

**Figure 1:**
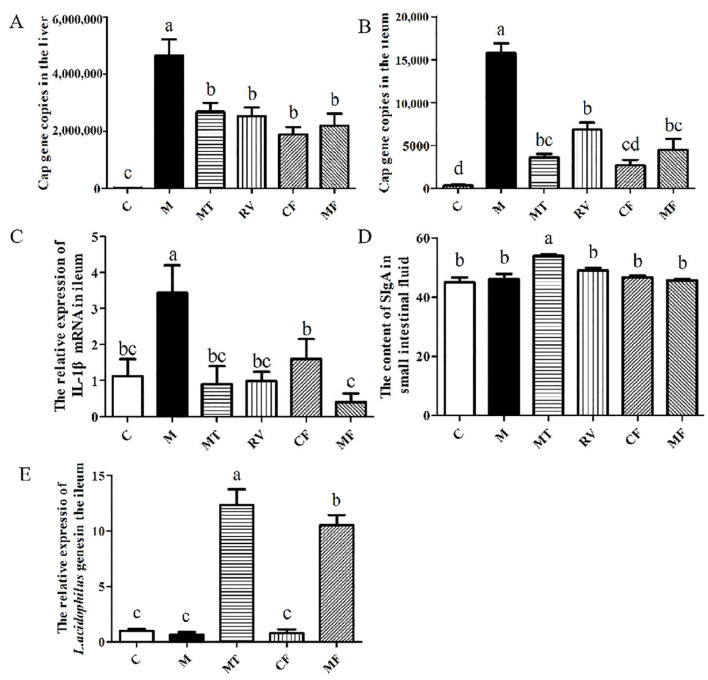
Effects of matrine fecal bacterial solution on the *Cap* gene copies in liver (**A**) and ileum (**B**), relative expression of *IL-1β* mRNA (**C**), SIgA content (**D**), and relative expression of *L. acidophilus* (**E**) in ileum. C: Control group; M: PCV2 infection model group; MT: Matrine group; RV: Ribavirin positive drug control group; MF: Matrine-treated fecal bacteria solution FMT group; CF: control fecal bacteria solution FMT group. The data are represented as the mean ± SD from at least three samples and three experiments that showed similar results by one-way ANOVA. Different letters (a–d) indicate significant differences between groups, *p* < 0.05.

The expression of the *Cap* gene in the liver and the ileum of each group was detected by qPCR at day 5 after the FMT. The results showed that compared with group M, the *Cap* gene copies in the liver of the MT, RV, CF, and MF groups were significantly reduced (*p* > 0.05) (Figure 1A). Compared with group M, the *Cap* gene copies in the ileum of the MT, RV, CF, and MF groups were also significantly reduced (*p* < 0.05). The *Cap* gene copies in the CF group were significantly lower than the RV group (*p* < 0.05), but there was no significant difference between the other groups (*p* > 0.05) (Figure 1B). The results showed that matrine-treated fecal bacterial solution or control fecal bacterial solution could both reduce the expression of the *Cap* gene in the liver and the ileum. Compared with the control group, PCV2 infection significantly increased the relative expression of *IL-1β* mRNA (*p* < 0.05). Compared with group M, the relative expression of *IL-1β* mRNA in the MT, RV, CF and MF groups was significantly decreased (*p* < 0.05). The relative expression of *IL-1β* mRNA in the MF group was significantly lower than that in the CF group (*p* < 0.05) (Figure 1C). The results showed that matrine-treated fecal bacterial solution or control fecal bacterial solution could reduce the relative expression of *IL-1β* mRNA, and that matrine-treated fecal bacterial solution had a more significant anti-inflammatory effect. The content of SIgA in the MT group was significantly higher than that in the C, M, RV, CF, and MF groups (*p* < 0.05) (Figure 1D). The results showed that PCV2 infection did not affect the content of SIgA, but that matrine could increase the content of SIgA, which was not related to intestinal flora. Compared with group C, PCV2 infection had no significant effect on the relative expression of the *L. acidophilus* gene (*p* > 0.05). The relative expression of the *L. acidophilus* gene of the MT and MF groups was significantly higher than that of the C, M, and CF groups (*p* < 0.05), and the MT group was significantly higher than the MF group (*p* < 0.05) (Figure 1E). The results showed that both MF and MT could significantly promote the colonization of *L. acidophilus* in the ileum of mice (*p* < 0.05), suggesting that the anti-PCV2 and anti-inflammatory effects of matrine may be related to *L. acidophilus*.

### 2.2. Identification of L. acidophilus

**Figure 2:**
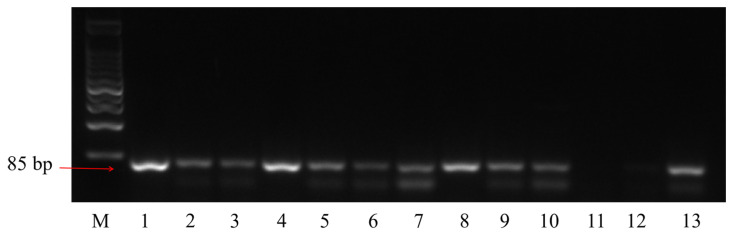
Identification of *L. acidophilus* by colony PCR.

The *L. acidophilus* in feces was isolated and cultured. The results of PCR identification (Figure 2) showed that the PCR product of strains 1, 4, and 8 was single band and that its size was in line with the theoretical value, which was used for further 16S rDNA sequencing identification. The 16S rDNA sequencing for the strain 1 failed due to its low template concentration, and the sequencing for strains 4 and 8 were successful.

**Figure 3:**
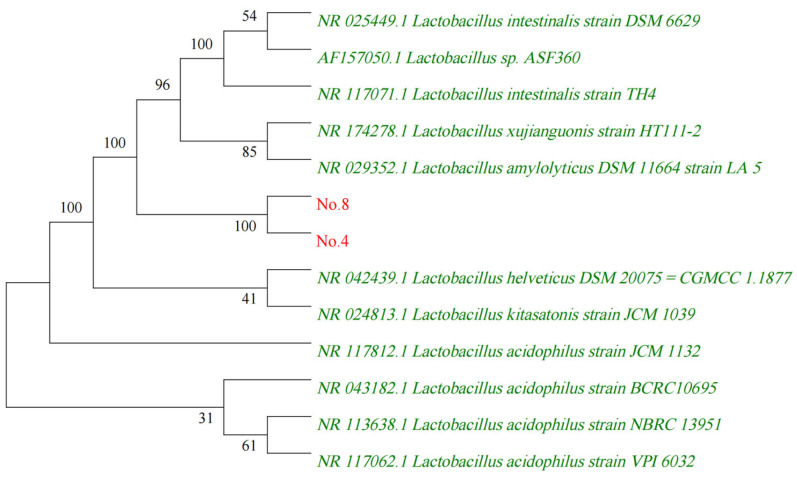
Phylogenetic tree based on 16S rDNA gene sequences.

The obtained 16S rDNA gene sequence data were submitted to GenBank BLAST search analyses, which yielded a strong homology of up to 98% with those of several cultivated strains of *lactobacillus*. The sequences of highly homologous *lactobacillus* were imported to construct a phylogenetic tree using MEGA 5.05 software (Figure 3). The pylogenetic tree showed the position of strain 4 and 8 within the radiation of the genus lactobacillus. The numbers at nodes (>50%) indicate support for the internal branches within the tree obtained by bootstrap analysis (percentages of 100 bootstraps). NCBI accession numbers are presented together with the strain name.

### 2.3. L. acidophilus Reduced the Relative Expression of IL-1β mRNA In Vivo and Had Anti-PCV2 Effects

**Figure 4:**
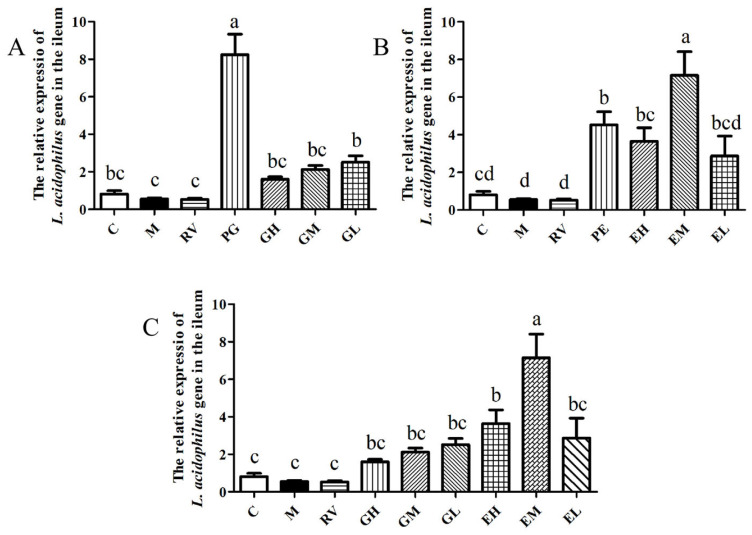
Effects of *L. acidophilus* transplantation on the relative expression of *L. acidophilus* in the ileum (**A**–**C**). C: Control group; M: PCV2 infection model group; RV: Ribavirin positive drug control group; PG: Preventive gavage group; GH: High dose gavage group; GM: Middle dose gavage group; GL: Low dose gavage group; PE: Preventive enema group; EH: High dose enema group; EM: Middle dose enema group; EL: Low dose enema group. The data are represented as the mean ± SD from at least three samples and three experiments that showed similar results by one-way ANOVA. Different letters (a–d) indicate significant differences between groups, *p* < 0.05.

After the mice were given *L. acidophilus*, the relative expression of the *L. acidophilus* gene in the ileum of each group was detected by qPCR. The results showed that compared with the M group, the relative expression of the *L. acidophilus* gene of the PG and GL groups was significantly increased (*p* < 0.05), and the GH and GM groups showed an increasing trend (Figure 4A). Compared with the M group, the relative expression of the *L. acidophilus* gene of the PE, EH, and EM groups was significantly increased (*p* < 0.05), and there was an increasing trend in the EL group (Figure 4B). As shown in Figure 4C, there was no significant difference in the relative expression of the *L. acidophilus* gene between the GH, GM, and GL groups and the EH and EL groups (*p* > 0.05), but it was significantly lower than the EM group (*p* < 0.05). The relative expression of the *L. acidophilus* gene in the enema group was higher than that in the gavage group. This shows that administration of different doses of *L. acidophilus* in mice by gavage and enema can lead to an increase in the relative expression of the *L. acidophilus* gene in the ileum, and that enema is more conducive to the colonization of *L. acidophilus*.

**Figure 5:**
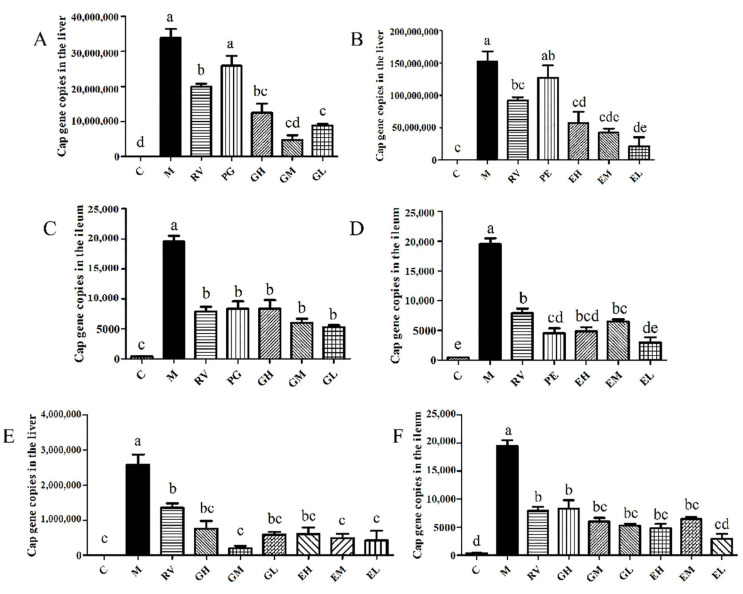
Effects of *L. acidophilus* transplantation on the *Cap* gene copies in the liver (**A**,**B**,**E**) and the ileum (**C**,**D**,**F**). C: Control group; M: PCV2 infection model group; RV: Ribavirin positive drug control group; PG: Preventive gavage group; GH: High dose gavage group; GM: Middle dose gavage group; GL: Low dose gavage group; PE: Preventive enema group; EH: High dose enema group; EM: Middle dose enema group; EL: Low dose enema group. The data are represented as the mean ± SD from at least three samples and three experiments that showed similar results by one-way ANOVA. Different letters (a–e) indicate significant differences between groups, *p* < 0.05.

After *L. acidophilus* transplantation, qPCR was used to detect the expression of the *Cap* gene in the liver. The results showed that compared with the M group, the *Cap* gene copies in the GH, GM, and GL groups were significantly reduced (*p* < 0.05) (Figure 5A). As shown in Figure 5B, compared with the M group, the *Cap* gene copies in the EH, EM, and EL groups were significantly decreased (*p* < 0.05). As shown in Figure 5E, there was no significant difference for the *Cap* gene copies between the gavage and the enema methods (*p* > 0.05). The results showed that after PCV2 infection, administration of *L. acidophilus* to mice could significantly reduce the *Cap* gene copies in the liver, and there was no significant difference between the different administration methods and the different doses (*p* > 0.05).

The expression of the *Cap* gene in the ileum of mice in each group was detected by qPCR. The results showed that compared with the M group, the *Cap* gene copies in *L. acidophilus* gavage treatment groups were significantly decreased (*p* < 0.05) (Figure 5C). As shown in Figure 5D, the *Cap* gene copies in the *L. acidophilus* enema treatment groups were significantly lower than that in the M group (*p* < 0.05), and the *Cap* gene copies in the EL group were significantly lower than that in the EM group (*p* < 0.05). Between the *L. acidophilus* gavage treatment groups and the enema treatment groups, only the EL group was significantly lower than the GH group (*p* < 0.05), and there were no significant differences in the ileum *Cap* gene copies between the other groups (*p* > 0.05) (Figure 5F). The results showed that administration of *L. acidophilus* by gavage or enema could significantly reduce the *Cap* gene copies in ileum. Within the dose range set in this experiment, 2.5 × 10^7^ CFU *L. acidophilus* by enema had a better effect on reducing the *Cap* gene copies in ileum.

**Figure 6:**
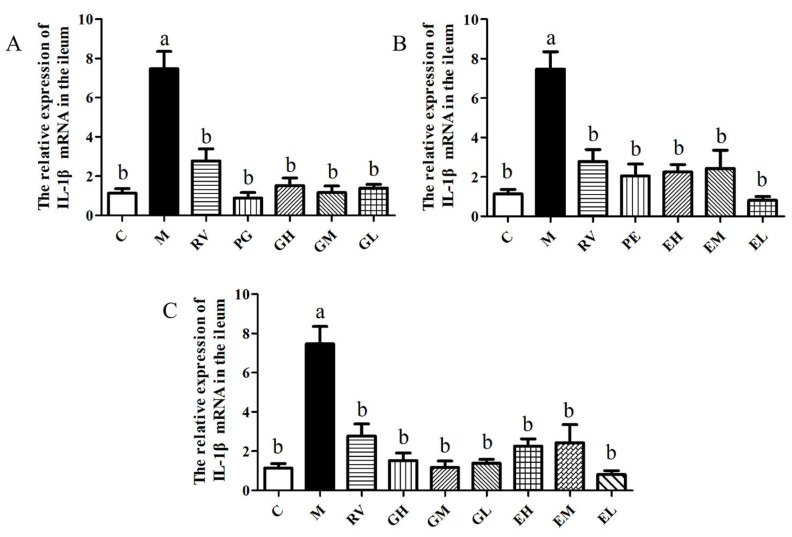
Effects of *L. acidophilus* transplantation on the relative expression of *IL-1β* mRNA in the ileum (**A**–**C**). C: Control group; M: PCV2 infection model group; RV: Ribavirin positive drug control group; PG: Preventive gavage group; GH: High dose gavage group; GM: Middle dose gavage group; GL: Low dose gavage group; PE: Preventive enema group; EH: High dose enema group; EM: Middle dose enema group; EL: Low dose enema group. The data are represented as the mean ± SD from at least three samples and three experiments that showed similar results by one-way ANOVA. Different letters (a,b) indicate significant differences between groups, *p* < 0.05.

The relative expression of *IL-1β* mRNA in the ileum of mice in each group was detected by qPCR. The results showed that the relative expressions of *IL-1β* mRNA in all *L. acidophilus* treatment groups were significantly lower than that of the M group (*p* < 0.05), and that there was no significant difference between the *L. acidophilus* treatment groups (*p* > 0.05) (Figure 6A–C). The results showed that *L. acidophilus* could significantly reduce the relative expression of *IL-1β* mRNA in the ileum of PCV2-infected mice by gavage or enema, and that there was no significant difference between the different methods and different doses (*p* > 0.05).

## 3. Discussion

PCV2 has caused huge economic losses to the pig industry in China and the world. Our previous study found that matrine has anti-PCV2 effect [14], but the mechanism of matrine against PCV2 is not clear. Niederwerder et al. found that the abundance of *L. acidophilus* in the intestine of pigs with severe clinical symptoms after PRRSV/PCV2 infection decreased [9]. Our previous study found that Matrine can regulate the composition of intestinal flora in normal mice and promote the colonization of *L. acidophilus*. Studies have shown that intestinal flora is an important target for Traditional Chinese Medicine to exert pharmacodynamic effects [24,25], suggesting that matrine may regulate intestinal flora, especially *L. acidophilus*, and exert anti-PCV2 effects.

The results of FMT showed that both control fecal bacterial solution and matrine-treated fecal bacterial solution could inhibit the replication of PCV2 in the liver and the ileum with no significant difference, indicating that FMT of control fecal bacterial solution can also play an anti-PCV2 role. Compared with the M group, the relative expression of ileal *IL-1β* mRNA in the CF and MF groups were significantly reduced, and the relative expression of ileal *IL-1β* mRNA in the MF group was significantly lower than that in the CF group, indicating that matrine-affected fecal bacterial solution has a stronger anti-inflammatory effect. This result suggested that matrine can play an anti-inflammatory role by regulating the intestinal flora.

In this study, the relative expression of *L. acidophilus* in the ileum of the MT and MF groups was significantly higher than that of other groups. Matrine and matrine-treated fecal bacterial solution had the effects of anti-PCV2 and reduced *IL-1β* mRNA expression. It is suggested that matrine can exert anti-PCV2 effects and reduce the relative expression of *IL-1β* mRNA by regulating the composition of intestinal flora, and matrine may exert this pharmacological effect by increasing intestinal *L. acidophilus*. The results of SIgA content showed that matrine could significantly increase the secretion of SIgA in PCV2-infected mice, and that there was no significant change in the MF group. The results showed that the antiviral effect of matrine has multiple targets, suggesting that intestinal flora might be one of the many targets of matrine against PCV2.

*L. acidophilus* is one of the most important probiotics. Previous studies have shown that *L. acidophilus* has antiviral [20] and anti-inflammatory [21] effects. In order to explore whether matrine can inhibit PCV2 by regulating the abundance of *L. acidophilus*, a strain of *L. acidophilus* was isolated and transplanted into PCV2-infected mice. The results showed that the administration of *L. acidophilus* by gavage or enema could significantly reduce the *Cap* gene copies in the liver and ileum and the relative expression of *IL-1β* mRNA in the ileum. At 5 days before PCV2 infection, gavage and enema of 10^8^ CFU *L. acidophilus* could significantly reduce the *Cap* gene copies in the ileum, but had no effect in the liver.

This result may be due to two reasons. Firstly, the anti-PCV2 effect of *L. acidophilus* is different in the liver and ileum. Studies have shown that *L. acidophilus* can exert antiviral effects through a variety of ways, including direct inhibition [26], regulation of the body’s immune status to resist virus, etc. [27]. The difference in the antiviral mechanism and the pathway of *L. acidophilus* may be the main reason for the difference in the anti-PCV2 effect of *L. acidophilus* in the liver and the ileum. Secondly, *L. acidophilus* treatment groups can reduce *Cap* gene copies in the liver, while the prevention groups have no anti-PCV2 effect. The difference between the treatment groups and the prevention groups is the length of *L. acidophilus* colonization in the gastrointestinal tract. The *L. acidophilus* in the prevention groups was colonized in the gastrointestinal tract for 15 days, while that in the treatment groups was only colonized for 5 days. The entry of *L. acidophilus* into the gastrointestinal tract may lead to changes in the overall composition and function of the intestinal flora, which may be the basis of the antiviral effect. However, after *L. acidophilus* entered the gastrointestinal tract for a long time, the composition and function of the intestinal flora gradually returned to normal under the action of self-regulation and repair, and the antiviral effect was weakened. This may be the main reason for the difference in anti-PCV2 between the treatment groups and the prevention groups.

There was no dose-dependent relationship between *L. acidophilus* and its anti-PCV2 effect. The results of *Cap* gene expression in the ileum showed that compared with the EH and EM group, the EL group had a stronger anti-PCV2 effect. The results of the relative expression of the *L. acidophilus* gene showed that the number of *L. acidophilus* in the low-dose groups and the high-dose groups was similar after 5 days, indicating that different doses of *L. acidophilus* had different proliferation processes in vivo. The level of the anti-PCV2 effect of *L. acidophilus* may be related to its proliferation process in vivo.

## 4. Materials and Methods

### 4.1. Compounds, Virus and KM Mice

**Figure 7:**
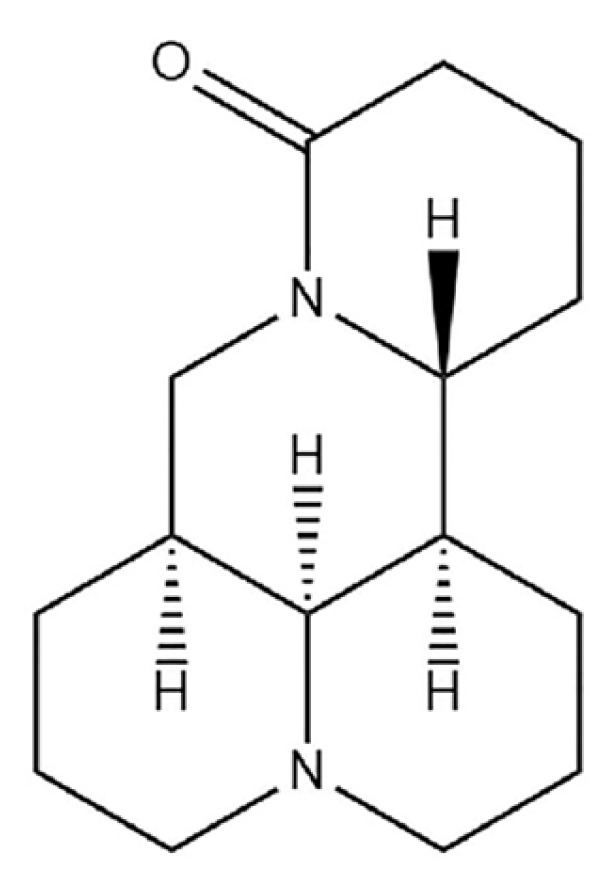
The chemical structure of matrine.

Matrine (batch number: ZLSC2018032020) was purchased from Nanjing Zelang Meditech Ltd., China, and its purity was 98% by HPLC. The chemical structure of matrine was shown in Figure 7. Ribavirin (Batch number: 36791-04-5) was purchased from Beijing Solarbio Technology Co., Ltd. (Beijing, China). The TCID_50_ of PCV2 was 10^−5.4^/0.1 mL. Female SPF KM mice were purchased from Beijing Vital River Laboratoty Animal Technology Co., Ltd. (Beijing, China, quality certificate number: 110011210115155452). All mice were raised in the Experimental Animal Management Center of Shanxi Agricultural University.

### 4.2. Preparation of Fecal bacteria Solution and FMT

**Figure 8:**
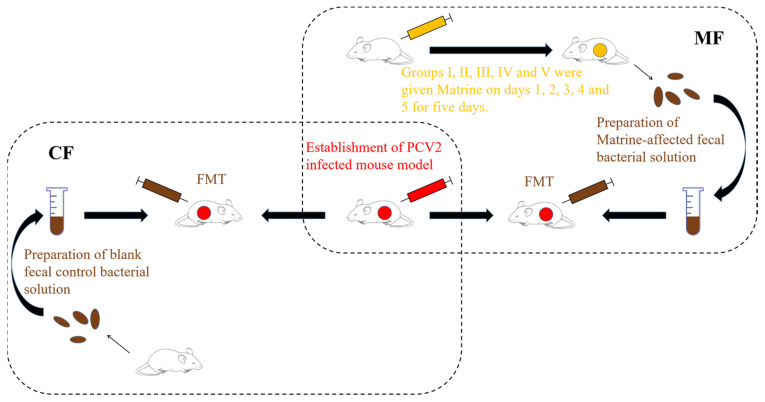
Preparation process of fecal bacteria solution.

A total of 60 SPF KM mice were randomly divided into 6 groups of 10. One group was the control group, and the feces were collected for the preparation of control fecal bacterial solution (CF). The other five groups were matrine administration groups (I, II, III, IV, V). Mice in these groups were intraperitoneally injected with 40 mg/kg of matrine at a dose of 0.2 mL/10 g twice a day for 5 consecutive days, and the procedure is shown in Figure 8 below. On the 6th day after the matrine administration, feces were collected, mixed with sterile saline, and filtered and centrifuged at 3000 rpm for 3 min for the isolation of matrine-treated fecal bacterial solution (MF). Then, 200 μL of fecal bacteria solution was taken via enema for FMT once a day for 5 days.

### 4.3. Effect of Matrine-Treated Fecal Bacteria against PCV2

A total of 60 SPF female KM mice were randomly divided into 6 groups of 10, including control group I, PCV2 infection model group (M), matrine group (MT), matrine-treated fecal bacteria solution FMT group (MF), control fecal bacteria solution FMT group (CF), and ribavirin positive drug control group (RV). Except for group C, mice in the other groups were intraperitoneally injected with a 0.5 mL medium containing PCV2. At day 5 after the PCV2 infection, mice in the MT and RV groups were intraperitoneally injected with 40 mg/kg of matrine and ribavirin, respectively, once a day for 5 days. Mice in the MF and the CF groups were given 200 μL of freshly prepared matrine-treated fecal bacterial solution and control fecal bacterial solution by enema, respectively, once a day for 5 days. The mice were sacrificed at day 6 after enema. The small intestinal fluid (small intestinal fluid at the junction of jejunum and ileum), liver, and ileum tissue samples were collected. The expression levels of the *Cap* gene in the liver and ileum, *IL-1β* mRNA in the ileum, and *L. acidophilus* gene in each group were determined by qPCR. The content of SIgA in the small intestinal fluid of each group was determined by ELISA.

### 4.4. The Expressions of CaI, IL-1β and L. acidophilus Genes Were Determined by qPCR

DNA of the liver, the ileum, and the ileum intestinal bacteria in each group was extracted following the instruction of Tianamp Genomic DNA Kit (Tiangen, Beijing, China), and used for analysis of the expression of the *Cap* gene and the relative expression of the *L. acidophilus* gene. Total RNA was extracted from the ileum according to the instructions of the TRIzol reagent (Takara, Dalian, China). cDNA was synthesized from the extracted RNA using a TaKaRa reverse transcription kit. The relative expression of *IL-1β* mRNA was determined using SYBR Green qPCR Master Mix from Bimake on an Applied Biosystems^®^ 7500 Real-Time PCR system.

The sequences of the primers used for detection of the *β-actin*, *IL-1β*, *Cap*, and *L. acidophilus* genes were shown in Table 1. The expression of the *Cap* gene was detected by qPCR using the extracted DNA as a template. The *Cap* gene recombinant plasmid was constructed previously in our laboratory. A serial dilution of the plasmid was used to construct a standard curve, so the expression of the *Cap* gene in the liver and the ileum was calculated as the copy number. The expression of *IL-1β* mRNA and *L. acidophilus* genes in the ileum was detected by qPCR using *β-actin* as an internal reference gene. The 2^−ΔΔCt^ method was used to calculate the relative expression of the gene.

### 4.5. The Content of SIgA in Small Intestinal Fluid Was Detected by ELISA

A total of 4 cm of intestinal tissue was taken at the junction of the jejunum and ileum in mice. The intestinal tract was cut longitudinally, and the intestinal mucosal layer was gently scraped with a sterile scalpel. The scraped samples were homogenized in an EP tube containing 0.5 mL PBS and centrifuged at 4000 rpm for 15 min, and the supernatant was extracted. The content of SIgA in the small intestinal fluid was detected using a mouse SIgA ELISA kit (Shanghai Enzyme Biotechnology Co., Ltd., Shanghai, China).

### 4.6. Isolation and Culture of L. acidophilus in Feces

Fresh feces of KM mice in the control group were collected to prepare the fecal bacterial solution, which was diluted ten times. A total of 500 μL of each dilution gradient of the bacterial solution was evenly coated on the LBS agar medium (Solarbio, Beijing, China) and anaerobically cultured at 37 °C for 48 h. The colonies with typical characteristics and vigorous growth of *L. acidophilus* were selected and cultured on LBS agar medium, anaerobic culture at 37 °C for 48 h, and repeated separation was performed 3 times. The single colony was isolated and cultured on the MRS agar plate (Solarbio, China) anaerobically for 48 h at 37 °C. This procedure was repeated for three times until the pure culture of the single strain was obtained.

### 4.7. Identification of L. acidophilus

The isolated colonies were used for colony PCR screening. PCR amplification was performed with *L. acidophilus* gene specific primers. The PCR reaction conditions were: 5 min pre-denaturation at 94 °C, 40 cycles of 30 s at 94 °C, 30 s at 60 °C, and 30 s at 72 °C, and a final 5 min extension at 72 °C. The amplified PCR products were subjected to agarose gel electrophoresis. A single band was identified as the amplified *L. acidophilus* gene fragment according to the predicted size. This DNA fragment was then purified from the gel and sent to Tsingke Biotechnology Co., Ltd. (Beijing, China) for 16S rDNA sequencing. The 16S rDNA gene sequences obtained were submitted to GenBank BLAST search analyses, and the highly homologous sequences were imported into MEGA software to construct a phylogenetic tree.

### 4.8. Anti-PCV2 Effect of L. acidophilus In Vivo

**Figure 9:**
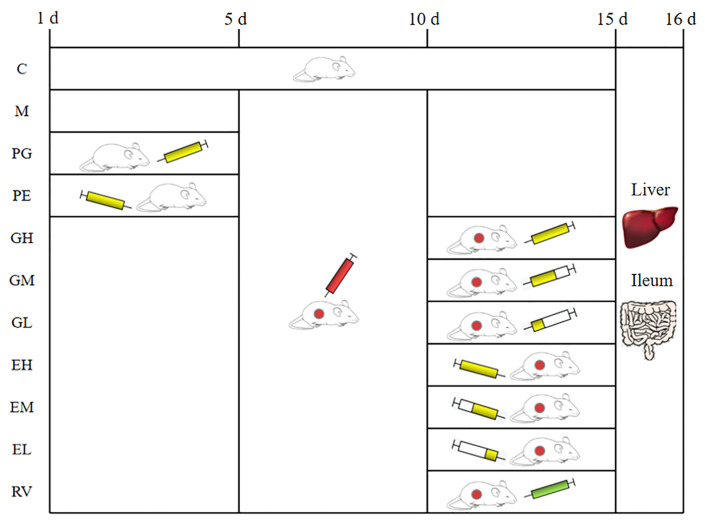
The specific treatment time for mice in each group. The blank area in the figure represents the normal feeding without any treatment.

A total of 88 SPF female KM mice were randomly divided into 11 groups of 8. They were divided into control group I (C), PCV2 infection model group (M), ribavirin positive drug control group (RV), preventive gavage group (PG), high dose gavage group (GH), middle dose gavage group (GM), low dose gavage group (GL), preventive enema group (PE), high dose enema group (EH), middle dose enema group (EM), and low dose enema group (EL). A total of 10^8^ CFU of *L. acidophilus* was pre-administered daily (once a day) to each mouse in the PG and the PE groups via gavage and enema. Totals of 10^8^, 10^6^, and 10^4^ CFU of *L. acidophilus* were administered to each mouse in the GH, GM, and GL groups via gavage, respectively. Totals of 10^8^, 5 × 10^7^ and 2.5 × 10^7^ CFU of *L. acidophilus* were administered to each mouse in the EH, EM, and EL groups via enema, respectively. The specific treatment times for the mice in different groups were shown in Figure 9. On the 16th day, the mice were culled and the liver and ileum samples were collected. The expression of the *Cap* gene in the liver and ileum as well as the relative expression of the *IL-1β* and *L. acidophilus* genes in the ileum were detected by qPCR.

### 4.9. Statistical Analyses

All data were analyzed using GraphPad Prism 5 (GraphPad Software, Inc., La Jolla, CA, USA) software. The Tukey method in one-way ANOVA was used to compare the differences between the groups. The lowercase letters between the groups indicated significant differences (*p* < 0.05).

## 5. Conclusions

In this study, we have demonstrated that administration of *L. acidophilus* significantly reduces the relative expression of the *IL-1β* mRNA and *Cap* gene copy numbers in the ileum of PCV2-infected mice. Matrine could upregulate the abundance of intestinal *L. acidophilus* of mice to exert its anti-PCV2 effect and inhibit PCV2-induced inflammatory response.

## Figures and Tables

**Table 1:** Primers used for qPCR.

Genes	Primer Sequences
*β*-actin	F: AGGGAAATCGTGCGTGACAT R: GGAAAAGAGCCTCAGGGCAT
*IL-1β*	F: GCCACCTTTTGACAGTGATGAGA R: GACAGCCCAGGTCAAAGGTT
*Cap*	F: GTCTACATTTCCAGCAGTTTG R: CTCCCGCCATACCATAA
*L acidophilus*	F: GAAAGAGCCCAAACCAAGTGATT R: CTTCCCAGATAATTCAACTATCGCTTA

## Data Availability

The data generated/analyzed during the current study are available from the corresponding author upon request.

## Abbreviations

Cap	Capsid
FMT	Fecal microbiota transplantation
KM	Kunming
*L. acidophilus*	*Lactobacillus acidophilus*
ORFs	Open reading frame
PCVAD	Porcine circovirus associated disease
PCV2	Porcine circovirus type 2
qPCR	Real-time Quantitative polymerase chain reaction
SIgA	Secretory immunoglobulin A.
